# Malignant granular cell tumour at the interventricular septum

**DOI:** 10.1093/icvts/ivac099

**Published:** 2022-04-07

**Authors:** Weng-Kin Lou, Heng-Wen Chou, Tom Wei-Wu Chen, Che-Yu Hsu, Jeng-Wei Chen

**Affiliations:** 1 Division of Cardiovascular Surgery, Department of Surgery, National Taiwan University Hospital, Taipei, Taiwan; 2 Graduate Institute of Clinical Medicine, College of Medicine, National Taiwan University, Taipei, Taiwan; 3 Department of Oncology, National Taiwan University Hospital, Taipei, Taiwan; 4 Graduate Institute of Oncology, College of Medicine, National Taiwan University, Taipei, Taiwan; 5 Division of Radiation Oncology, Department of Oncology, National Taiwan University Hospital, Taipei, Taiwan

**Keywords:** Granular cell tumour, Cardiac tumour, Interventricular septum, Surgery

## Abstract

Granular cell tumours are usually benign with a 1–2% incidence of malignancy. They are less sensitive to radiotherapy and chemotherapy and are treated by surgical excision. We report a case of a malignant granular cell tumour located at the interventricular septum.

## INTRODUCTION

Granular cell tumour (GCT) was first described by a Russian pathologist, Abrikossoff, in 1926 [1]. Further studies reported that it is predominantly derived from the neural tissue, with few exceptions. GCT is believed to be mostly benign with a 1–2% incidence of malignancy [2]. It is less sensitive to radiotherapy and chemotherapy, with surgical excision being the most effective therapy to treat this malignancy. We present a rare case of a potential highly metastatic malignant cardiac GCT located at the interventricular septum (IVS).

## CASE REPORT

**Figure 1:**
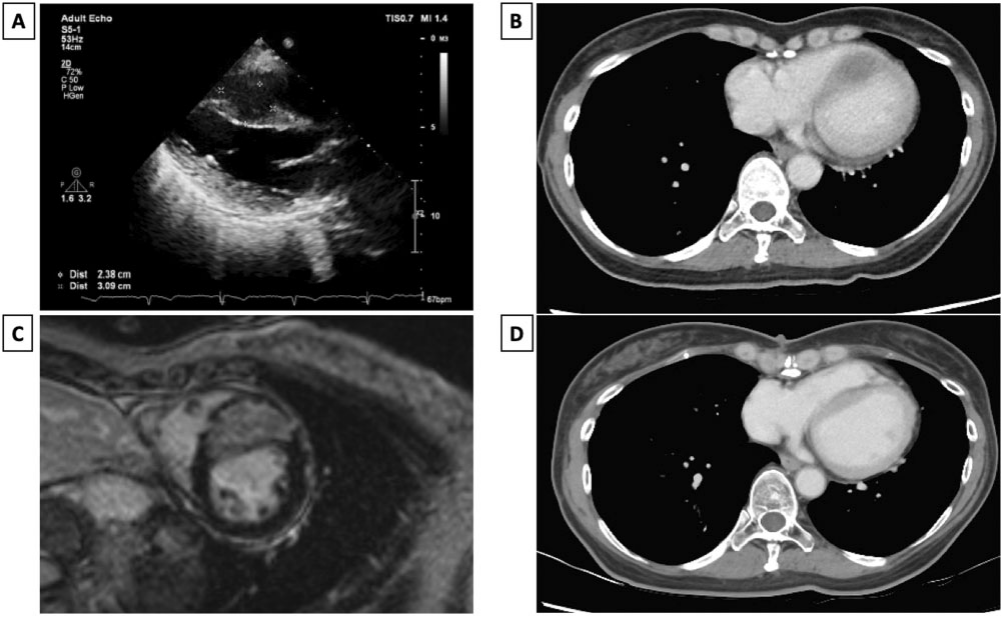
(**A**) Echocardiography shows a 3.1 × 2.2-cm tumour over the interventricular septum (**B**) Preoperative axial contrasted chest computed tomography shows a hypodense tumour at the interventricular septum. (**C**) Cardiac magnetic resonance imaging shows an interventricular mesocardium mass with delayed enhancement and increased T1 value. (**D**) Postoperative axial contrasted chest computed tomography at the 15-month follow-up.

A 48-year-old woman was diagnosed with a right thigh malignant GCT (8.2 cm) and underwent complete margin-free tumour resection 6 years ago. No chemotherapy or radiation therapy was administered after the first surgery. At the 3-year follow-up, a 0.5 cm right lower lobe lung tumour was observed on chest radiography, which enlarged to 2 cm in 2 years. She underwent a second surgery through a video-assisted thoracoscopic right lower lobe wedge resection, and the histopathology confirmed malignant GCT. After the surgery, she was started on metronomic oral cyclophosphamide. Follow-up computed tomography revealed an abnormal protruding IVS and a 1.9-cm hypodense oval tumour located in the IVS, which increased to 3.1 cm at the ninth-month serial images follow-up (Fig. 1B). On cardiac echography, the mass was hypoechoic and well capsulated (Fig. 1A). Magnetic resonance imaging revealed an interventricular mesocardial mass at the anteroseptal segments of the left ventricular myocardium, with delayed enhancement and increased T1 value (Fig. 1C).

**Figure 2:**
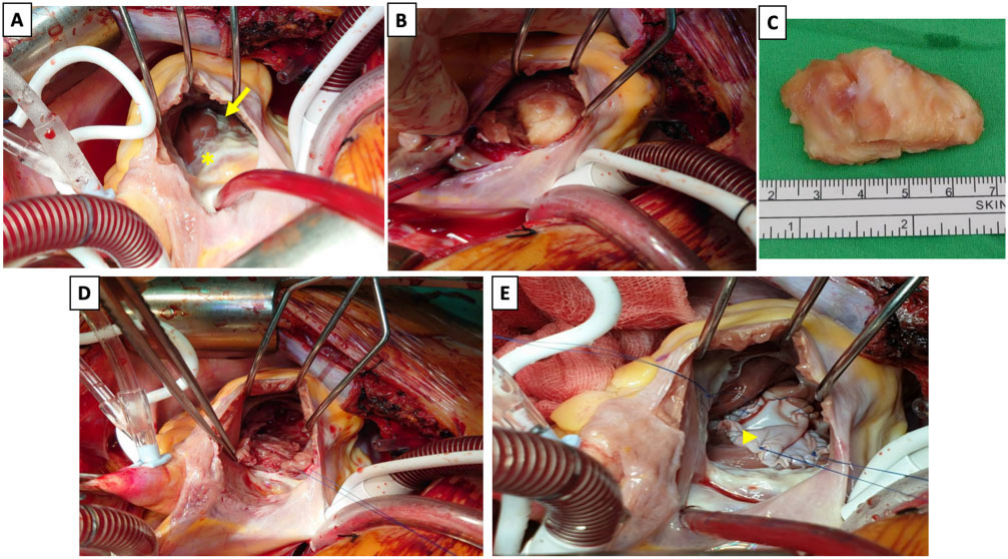
The intraoperative findings demonstrate (**A**) a whitish tumour (arrow) beneath the trabeculae in the interventricular septum (asterisk: septal leaflet of the tricuspid valve). (**B**) After partially excising the endocardium, the tumour is well-exposed. (**C**) A 4.7 × 2.8 × 2.8-cm tanned and elastic tumour. (**D** and **E**) The tumour bed is reinforced using a bovine pericardium patch (arrowhead). (A colour version of this figure appears in the online version of this article)

She underwent a cardiac tumour excision through the right atrium approach. After median sternotomy, the cardiopulmonary bypass was set up with ascending aorta and bicaval venous cannulation. After atriotomy, stay sutures are placed on the edges to open the right atrium. A well-defined yellow-whitish tumour was noted beneath the right ventricular trabeculae by retracting the anterior and septal leaflets of the tricuspid valve (Fig. 2A). After partially excising the trabecula and splitting the myocardium layer of the IVS, the tumour could be explored (Fig. 2B); it was completely removed, and the IVS layer was preserved. To reinforce the IVS of the right ventricular side, a bovine pericardial patch was sutured to the tumour bed (Fig. 2E). The histopathology confirmed malignant GCT with R1 resection. The histopathological characteristics of tumours were reviewed from 3 sites: bone, lung and heart, which showed the same pattern and high suspicion of metastasis. She received adjuvant radiotherapy for the mesocardium tumour bed. She was healthy after 18 months of follow-up. Follow-up computed tomography and echocardiography demonstrated no local tumour recurrence and normal heart function.

## DISCUSSION

GCT is a rare soft tissue tumour originating from Schwann cells. Malignant GCTs are predominantly located on the head, neck and trunk [1]. Distant metastasis is reported in half of the cases, and local recurrence is usual [1]. Only a few GCT cases demonstrate cardiac involvement, mostly observed in the ventricular myocardium, and the patients are asymptomatic [3]. However, some patients with conduction system involvement, for example, the sinoatrial node, may present with sudden death [4]. The advanced GCTs with diffuse myocardium infiltration may also compromise the coronary arteries, resulting in myocardial infarction [5]. Many studies reported successful cases of extra-cardiac malignant GTC excision. However, less information regarding the cardiac GCT excision exists. Gualis *et al.* [5] reported that one patient with advanced cardiac GCT was treated with a heart transplant. We reported a rare case of cardiac malignant GCT. Based on our experience, this tumour was dense and sparsely invaded the surrounding tissues. Although total *en bloc* resection of the heart is impossible, it can be easily separated from the normal myocardium to obtain R1 resection in its early stage. Combined with adjuvant radiotherapy and chemotherapy, the prognosis of cardiac malignant GCTs was acceptable.

**Conflict of interest:** none declared.

### Reviewer information

Interactive CardioVascular and Thoracic Surgery thanks Nikolay O. Travin, Alberto Repossini and the other anonymous reviewer(s) for their contribution to the peer review process of this article.
